# Antibiotic dry buffalo therapy: effect of intramammary administration of benzathine cloxacillin against *Staphylococcus aureus* mastitis in dairy water buffalo

**DOI:** 10.1186/s12917-020-02410-7

**Published:** 2020-06-12

**Authors:** Jacopo Guccione, Luigi D’Andrea, Antonella Pesce, Fausto Toni, Giuliano Borriello, Caterina Salzano, Francesco Diuccio, Massimo Pascale, Paolo Ciaramella

**Affiliations:** 1 Department of Veterinary Medicine and Animal Production, University of Napoli Federico II, Via Delpino 1, 80137 Naples, Italy; 2 Istituto Zooprofilattico del Mezzogiorno, Via A. Jervolino 19, Caserta District, 81100 Tuoro, Italy; 3 Zoetis, Italia s.r.l., Via Andrea Doria 41M, 00192 Rome, Italy; 4 Veterinary practitioner, Caserta, Italy

**Keywords:** Antibiotic dry therapy, Mastitis, Water buffalo, *Staphylococcus aureus*, Benzathine cloxacillin

## Abstract

**Background:**

Mastitis is one of the most costly diseases in Mediterranean buffalo (MB). At present, just a few specific antibiotics registered for this dairy specie have been synthetized. Efficacy of an antibiotic dry buffalo therapy (aDBT) against *Staphylococcus aureus* (*S. aureus*) mastitis, based on intra-quarter administration of 600 mg of benzathine cloxacillin, have been evaluated for the first time. Eighty MB’s quarters received a drying-off therapy (aDBT-group) and 80 were left untreated (no-aDBT-group). They were sampled at drying-off (pre-treatment) and at the resumption of milking [< 10 days in milk (DIM)]. *Fresh calver mastitis rate*, *dry period new mastitis rate*, *dry period cure rate*, and *persistent mastitis rate* were calculated for clinical monitoring. Overall proportion of positive quarters/animals, quarters affected by mastitis or intramammary infections (IMI), effects on somatic cell count (SCC) and milk yield were also assessed.

**Results:**

An inter-group difference (aDBT vs. no-aDBT) was recorded for all the indexes considered. An intra-group (drying-off vs. < 10 DIM) difference was detected in aDBT-group regarding the proportion of positive-cultured quarters and animals. Concerning the latter, an inter-groups difference was also recorded at second sampling. No clinical mastitis due to the *S. aureus* was observed. Regarding the subclinical ones, a higher intra-group difference was observed in aDBT than no-aDBT group, while an inter-group difference was recorded at second sampling. No protective effect was observed against IMI. SCC showed an inter-group difference at second sampling, while none difference was instead detected for milk yield.

**Conclusions:**

The effects against *S. aureus* mastitis of benzathine cloxacillin administration at drying-off were assessed for the first time in MB. Its use shows encouraging results in reducing the proportion of mastitis and positive animals at the resumption of the lactation.

## Background

Mastitis can be considered one of the most expensive diseases for Mediterranean buffalo (MB) farmers and the resulting dairy food chain [1, 2]. Based on what has been reported in the literature, several contagious, (e.g. *Staphylococcus aureus, Streptococcus agalactiae*), environmental (e.g. *Streptococcus dysgalactiae, Escherichia coli*) and opportunist (e.g. Coagulase-Negative Staphylococci*, Streptococcus pluranimalium*) bacteria were recognized as mastitis-causing in MB [3–5]. Although recent studies show a decrease of milk yield in buffalo herd affected by mastitis-causing bacteria and a consequent farm’s gain reduction, information on their epidemiology within the herd, clinical findings related, and strategies for integrated problem management are still rare and incomplete compared to cow medicine [5–7]. According to our previous studies, *Staphylococcus aureus* (*S. aureus*) can be considered one of the most representative gram-positive bacteria causing in-udder disorders and alterations of mammary secretion in MB. Indeed, *S. aureus* has been associated with a high dry period new infection rate within the herd (up to 55.5%), a significant increase of SCC values, a decrease of milk yields, quality, and farm’s economic incomes when it causes subclinical (SCM) and clinical mastitis (CM) [2, 5, 6, 8].

In MB as described in cow, a beginning of lactation with a low prevalence of intramammary infection (IMI) is a desirable prerequisite to ensure optimal milk productions and udder health during the following milking time [7, 9, 10]. During the dry period, the goal of udder-health management programs is to minimize the number of infected quarters at calving and the chances of increases of SCC in early lactation stages [9, 10]. Both in MB and cow’s lactation stages, this is recognized as a critical time for mastitis control [4, 9, 10], nevertheless only for the second one, several strategies have been developed to control the effects of mastitis-causing bacteria. In this respect, antimicrobial dry therapy represented a cornerstone of any mastitis control program over the years. As reported in the literature, in-teats infusions of slow-release antibiotic preparations should cure the existing IMIs and protect the cows by new infections that could originate from this period [11]. As the prevalence of persistent contagious pathogens declined over-time, the need for a blanket dry cow therapy consequently decreased [11, 12]. The change has been also promoted by several controversies concerning the complex, and sometimes unclear, cause and effect relationship existing between this type of therapy and the appearance of antibiotic-resistant microorganisms [11, 12]. As a result, the attention has been focused on different strategies such as the selective dry cow therapy: a pathogen-, cow- and herd-specific treatment method [7, 12, 13]. Nevertheless, even the latter cannot be considered as a universal remedy because herd characteristics and management can deeply limit its use at an individual-cow level [11].

Unfortunately, MB’s medicine suffers from poor scientific knowledge compared to the bovine one, regarding this relevant topic [5], and at the best of the authors’ knowledge, no studies investigated the antibiotic’s clinical efficacy in MB at drying-off, so far. Even though some MB’s farms perform antibiotic dry buffalo therapy (aDBT), there is no evidence proving the effectiveness of this strategy for bacteria control in these ruminants characterized by a dry-off period even longer than cows (~ 4 months) [5]. Although on one side aDBT protocols are performed by veterinary practitioners merely transferring knowledge arising from bovine medicine, on the other one the issue becomes even more serious considering that just a few antibiotics are specifically synthesized for MB [13]. The legal inclusion of buffalo within the bovine species by the EU since 2009 [14], promoted the use of drugs manufactured for bovine in these ruminants, without scientific proves of their efficacy [13]. For this reason, a sensible reduction of emphasis on the responsible use of antibiotics has been observed as the main effect.

Based on the previous considerations, the present manuscript aims to investigate for the first time the clinical outcomes originating from a dry buffalo therapy against *S. aureus* mastitis. Therefore, the effects of an intramammary administration of benzathine cloxacillin have been defined through (i) the calculation of clinical indexes for mastitis monitoring, (ii) the assessment of the effects on udder health and (iii) milk yield.

## Results

### Farm and animals

Out of eight farms investigated, the selected one was the only meeting the eligibility criterion. In this farm, no differences in all the aspects of herd management practices were observed during the two study years (e.g. therapeutic protocols for mastitis, milking routine, feeding and housing system, dairy workers, etc.).

Regarding the overall number of MBs investigated, it represents the result of restrictive eligible criteria used, noticeably reducing the freedom of animal selection. Moreover, the enrolment originated from all MBs dried-off during the first useful season after farm detection (summertime), and thereby prolonging beyond that period the recruitment would have been achieved a sampling population even more over-time distributed and without the possibility to reduce possible seasonal influences on the results (e.g. different environmental conditions).

None MB (both in aDBT and no-aDBT group) shows neither signs of milk leaking in the first week or swollen quarters and teat damage during the entire dry period.

### Bacteriological milk culture

During the investigation, 640 quarters milk samples have been collected and submitted to SCC analysis and BMC (i.e. 40 MBs × 4 quarters sampled 2 times × 2 samplings time). A full agreement was always found for *S. aureus* identification when double samples have been cultured.

Type and proportion of the in-udder pathogens isolated at the two samplings times and in both the groups are reported in detail in Table 1. Briefly, *S. aureus was* the most commonly isolated in both the samplings and groups, followed by coagulase-negative staphylococci (CNS) at drying-off, and *E. coli* at first sampling of the new lactation. The overall percentage of other udder pathogens detected (not-*S. aureus*) was 19.8% (16/80) in aDBT group and 22.4% (18/80) in no-aDBT one at drying-off, while it was 52.3% (42/80) and 58.4% (47/80) in the same groups within 10 DIM, respectively. Negative-culture status was observed in 41.2% (33/80) and 11.2% (9/80) of the samples in aDBT and no-aDBT groups at drying-off, respectively; the 13.7% (11/80) and 27.5% (22/80) were instead recorded in the same groups within 10 DIM.

**Table 1:** Overall bacteriological milk culture results originating from the aDBT and the no-aDBT groups samples at drying-off and at the resumption of milking (< 10 DIM). Percentages represent the proportion calculate on the total number of bacteriological examinations

Pathogens	Gram stain	***Sampling at drying-off***						***First sampling of the new lactation***					
		Overall No.	%	No. in aDBT group	%	No. in no-aDBT group	%	Overall No.	%	No. in aDBT group	%	No. in no-aDBT group	%
*Staphylococcus aureus*	Gram+	73/160	45.6	40/80	50.0	33/80	41.2	44/160	27.5	18/80	22.5	29/80	36.3
*Coagulase-negative staphylococci*	Gram+	17/160	10.6	9/80	11.2	8/80	10.0	22/160	13.7	13/80	16.2	9/80	11.2
*Streptococcus agalactie*	Gram+	3/160	1.9	3/80	3.7	/	/	2/160	1.2	1/80	1.2	1/80	1.2
*Streptococcus dysgalactie*	Gram+	/	/	/	/	/	/	1/160	0.6	/	/	1/80	1.2
*Streptococcus thoralensis*	Gram+	3/160	1.9	3/80	3.7	/	/	/	/	/	/	/	/
*Streptococcus uberis*	Gram+	2/160	1.2	1/80	1.2	1/80	1.2	2/160	1.2	/	/	2/80	2.5
*Streptococcus* spp.	Gram+	/	/	/	/	/	/	11/160	6.9	5/80	6.2	6/80	7.5
*Enterococcus faecalis*	Gram+	/	/	/	/	/	/	1/160	0.6	/	/	1/80	1.2
*Aerococcus viridans*	Gram+	8/160	5.0	/	/	8/80	10.0	2/160	1.2	1/80	1.2	1/80	1.2
Other *bacteria*	Gram+	13/160	8.1	7/80	8.7	6/80	7.5	28/160	17.5	20/80	25.0	8/80	10.0
*Escherichia coli*	Gram-	1/160	0.6	/	/	1/80	1.2	30/160	18.7	16/80	20.0	14/80	17.5
*Aeromonas* spp.	Gram-	/	/	/	/	/	/	13/160	8.1	6/80	7.5	7/80	8.7
*Klebsiella oxytica*	Gram-	/	/	/	/	/	/	5/160	3.1	/	/	5/80	6.2
Other *bacteria*	Gram-	1/160	0.6	/	/	1/80	1.2	10/160	6.2	5/80	6.2	5/80	6.2
Negative		42/160	26.2	33/80	41.2	9/80	11.2	33/160	20.6	11/80	13.7	22/80	27.5

*aDBT* antibiotic dry buffalo therapy, *DIM* days in milk, *No.* number, *%* percentage

### Clinical indexes for mastitis monitoring

Concerning the *fresh calver mastitis rate, a* significant difference (*P <* 0.001) was recorded between the proportions detected in the aDBT (1.3%, 1/80) and in the no-aDBT groups (20.0%, 16/80). An additional inter-group difference (*P <* 0.01) was observed regarding the *dry period new mastitis rate* due to *S. aureus.* Indeed, no new cases were observed in the treated group (0/13), while they were found in the untreated one with a proportion of 44.4% (8/18). Regarding the *dry period cure rate* and *persistent mastitis rate* caused by *S. aureus, a* significant inter-group difference was recorded for the first (*P <* 0.0001) and the second (*P <* 0.05) parameter mentioned. Indeed, a proportion of cured quarters of 97.4% (37/38) and 41.7% (10/24) was found in aDBT and no-aDBT group, respectively; for the second index, the proportion of not-cured quarters was instead of 2.6% (1/38) in aDBT group and 20.8% (5/24) in the no-aDBT.

### Udder health status

The overall proportion of *S. aureus* positive-cultured quarters at drying-off was 50.0% (40/80) in aDBT group and 41.2% (33/80) no-aDBT one; the same parameter evaluated within 10 DIM showed a proportion of 22.5% (18/80) and 36.3% (29/80), respectively. A statistically significant intra-group difference has been observed in the treated group (*P <* 0.01), while an inter-group difference was found for the sampling within 10 DIM (*P <* 0.01).

The proportion of quarters affected by *S. aureus* infection with different health status are laid down in detail in Table 2. Briefly, neither in aDBT or no-aDBT group CM due to the *S. aureus* were never observed during the entire investigation. Regarding SCM, a statistically significant intra-group difference has been observed between the two samplings time both in the treated (*P <* 0.01) and untreated group (*P <* 0.05); an inter-group difference has been also detected at milk resuming (*P <* 0.001) (Table 2). The RR of mastitis calculated within 10 DIM in aDBT group (1/79) than no-aDBT group (16/64) was 0.06 (95% CI = 0.01–0.46; *P <* 0.01). In all the quarters affected by SCM, *S. aureus* was always isolated in mono-infection.

**Table 2:** Different quarters’ health status in pluriparous Mediterranean buffalos affected by *S. aureus* infections in aDBT and no-aDBT groups. Samplings were performed at drying-off and at the resumption of milking (< 10 DIM). Percentages represent the proportion calculate on the total number of quarters enrolled

Clinical *status*	*Sampling at drying-off*				*First sampling of the new lactation*			
	No. in aDBT group	%	No. in no- aDBT group	%	No. in aDBT group	%	No. in no-aDBT group	%
CM	/	/	/	/	/	/	/	/
SCM	38/80^c^	47.5	25/80^a^	31.2	1/80^d,e^	1.3	16/80	20.0^b,f^
IMI	2/80^e^	2.5	8/80	10.0	17/80^f^	21.3	13/80	16.2

*aDBT* antibiotic dry buffalo therapy, *DIM* days in milk, *No.* number, *aDBT* antibiotic dry buffalo therapy, *%* percentage, *CM* clinical mastitis, *SCM* subclinical mastitis, *IMI* intramammary infection; ^a,b^*P* < 0.05; ^c,d^*P* < 0.01; ^e,f^*P* < 0.001

Regarding IMI, a statistically significant intra-group difference has been observed only in the treated group (*P <* 0.001) (Table 2). At drying-off, *S. aureus* has been always detected in co-infection in quarters affected by IMI. Furthermore, at the first sampling of the new lactation, it was instead detected as responsible of co-infection in 58.8% of cases (10/17) in a-DBT group, and in 61.5% (8/13) in no-aDBT group. All co-infections were always caused by not-recognized udder specific pathogens in buffalo (i.e. *Lactobacillus* 7/28, *Geobacillus toebii* 5/28, *Kocuria rosea* 8/28, *Kocuria kristinae* 8/28).

Instead, the overall proportion of udders of buffaloes positive for *S. aureus* at drying-off was 75.0% (15/20) and 65.0% (13/20) in aDBT and no-aDBT group, respectively. The same parameter evaluated at the first sampling of the new lactation gave a proportion of 40.0% (8/20) and 65.0% (13/20), respectively. A statistically significant intra-group difference has been observed only in the treated group (*P <* 0.001). Regarding the aDBT group, the 65% of the animals (13/20) showed at least one quarter with SCM at dry-off (i.e. 1 MB with 1 quarter affected by SCM, 3 MBs with 2 quarters, 5 MBs with 3, and 4 MBs with 4). In this group, the only case of SCM recorded at milking resumption belonged to one subject that cured 3 of the 4 quarters affected.

### SCC and milk yield

Data regarding Log10 SSC and milk yield recorded in both the samplings, either for aDBT or for no-aDBT, are reported in detail in Table 3. Briefly, regarding the overall average of SSC, no significant, differences were observed between the two groups at drying-off, while it was found at second sampling (aDBT vs. no-aDBT, *P <* 0.01). Moreover, intra-group differences have been detected within both within the aDBT group (*P <* 0.0001) and the no-aDBT one (*P <* 0.001). If quarters affected by mastitis due to *S. aureus* are discharged from the analysis, an inter-group significant difference is observed only in the first sampling in the new lactation (*P <* 0.05); an intra-group difference was detected only for aDBT animals (*P <* 0.05).

**Table 3:** Overall averages of somatic cells count and milk yields as well as data relative to negative and IMI buffaloes belonging to aDBT and no-aDBT groups at the two samplings time (dry-off and < 10 DIM)

Samplings	aDBT		no-aDBT	
	Overall	Neg. and IMI	Overall	Neg. and IMI
**SCC (Log**_**10**_**cells/mL)**				
*Drying-off*	5.7 ± 1.1^g^	5.2 ± 0.8^A^	5.8 ± 0.7^e^	5.5 ± 0.7
*< 10 DIM*	5.0 ± 0.7^c,h^	4.9 ± 0.6^a,B^	5.4 ± 0.6^d,f^	5.2 ± 0.6^b^
**Milk yield (L/d)**				
*Drying-off*	0.4 ± 0.2^α^	0.4 ± 0.1^α^	0.5 ± 0.2^α^	0.5 ± 0.2^α^
*< 10 DIM*	5.3 ± 1.5^β^	5.2 ± 1.4^β^	5.2 ± 1.1^β^	5.3 ± 1.1^β^

*aDBT* antibiotic dry buffalo therapy, *SCC* somatic cell count, *Log* logarithm, *mL* millilitres, *Neg*. negative, *IMI* intramammary infection, *DIM* days in milk, *L* litres, *d* days. Statistical significances for SCC are reported as: ^a,b^*P* < 0.05, ^A,B^*P* < 0.05, ^c,d^ P < 0.01; ^e,f^ P < 0.001; ^g,h^*P* < 0.0001. Statistical significances for milk yields have to be considered within the columns: ^α,β^*P* < 0.0001

Finally, regarding the daily mean milk yield, no significant inter-group differences were observed in both the samplings. Intra-group differences were instead found both within the aDBT group (*P <* 0.0001) and within the no-aDBT one (*P <* 0.0001). If quarters affected by mastitis are discharged from the analysis, no significant inter-group differences are assessed; they can be instead found both for the aDBT group (*P <* 0.0001) and for the no-aDBT one (*P <* 0.0001).

## Discussion

Estimation under field conditions of aDBT effects against *S. aureus* mastitis with supposed dry period origin is an interesting challenge in a dairy species like MB where the knowledge regarding management of mastitis are almost rare [1]. The complete lack of previous knowledge concerning the effect of an aDBT forced the authors to careful and methodical approach for the evaluation of the outcomes resulting from an antibiotic synthesized for dairy cows in origin. Therefore, the intended goal has been achieved verifying the tolerance of the animals to the product, detecting udder’s clinical findings, recording the milk quality and yields, as performed in other previous studies [5, 10].

The responsible use of antibiotics and the concerns regarding potentially resulting bacterial resistance, affect both veterinary and human medicine [15]. Therapy at dry-off is one of the most important herd management areas where the previous concepts should be applied, representing a true challenge for the next future. Only the uphold of principles such us responsible use of antibiotics, analysis of past prescribing practices and outcome, combined with robust clinical research evidence can represent a starting point for rational and efficient use of antibiotics in MB as much as in cows [15, 16]. To interpret these principles, the present study provides evidence on clinical findings associated with aDBT allowing some scientific considerations never conceived of before in MB’s mastitis management. Our investigation may be considered a starting point to build the essential basic knowledge, to understand the importance of antibiotics rational use even in this specie, to promote further studies investigating the effects on udder health of selective/targeted treatments. As described in cow medicine [15, 16], the approach to aDBT cannot be only focused on antibiotic use but should be supported by a complete knowledge of factors: social environment (e.g. attitude to antibiotic use), pharmacological environment (e.g. available products), and physical environment (e.g. spaces for the animals, management). According to the authors’ opinion, the lack of relevant knowledge may represent a strong point since the scientific community may look to bovine medicine as an example, avoiding to make analogous mistakes and taking only the best experiences and conclusions. Indeed, in the last years, the continuous transferring of knowledge from bovine herd management practices to MB is enabling the appearance of analogous pathologies [1, 17] and herd problems [18], emphasizing the necessity to improve knowledge regarding animal health and farm profitability [2, 19, 20].

According to the label’s indications [21], the bactericidal in action and not destroyed by staphylococcal beta-lactamase antibiotic used during the study was characterized by an off-white stable suspension of cloxacillin as the benzathine salt in a long-acting mineral oil base, prepared under sterile conditions. Its use in dairy cow has been recommended for routine use at drying-off, to treat existing IMI and to provide prolonged protection against new infections during the dry period [21]. Orbenin Extra® is described as active against Gram-positive mastitis-causing bacteria including *Trueperella pyogenes*, streptococci (e.g. *Str. agalactiae, Str. dysgalactiae, Str. uberis*), penicillin-resistant and sensitive *staphylococci* (e.g. *S. aureus*). The present investigation has been focused on clinical outcomes originating from an aDBT against *S. aureus*. Indeed, in our previous studies, this microorganism has been detected as the most representative Gram-positive in-udder pathogens, related with high contagiousness, causing mastitis and economic losses due to its negative influence on milk quality and yield in dairy MB [2, 5, 6]. Considering the overall BMC, a high-frequency isolation of *S. aureus* followed by CNS has been recorded in both the groups (Table 1). Analogous behaviour has been described both in MB [6] and cow herds [22], where frequencies of isolation of CNS and *Streptococci* were indeed considerably lower in herds positive for *S. aureus*. Moreover, as reported also cows [23], an evidence of its high contagiousness and pathogenicity can be also considered the high mono-infection isolation frequency observed both in treated and untreated groups during both the samplings.

The main goal of the present study was to verify the effects on *S. aureu*s mastitis with likely dry period origin by means of calculation of clinical indexes. Nevertheless, to avoid a potential bias related to a fix classification of udder health status on the results, the authors during analysis evaluated before the overall proportion of animals/quarters infected independently from their udder health status. The observed results regarding (i) a significant decrease of positive buffaloes and quarters in aDBT group (drying-off vs. < 10 DIM; *P <* 0.05 and *P <* 0.01, respectively), (ii) the inter-group difference for the same parameter at milking resumption (aDBT vs. no-aDBT, *P <* 0.01), and (iii) the overall reduction of mono-infection cultured within the aDBT group (drying-off vs. < 10 DIM, *P <* 0.0001) may provide a first interesting indication of benzathine cloxacillin’s effects in reducing and managing *S. aureus* udder’s infections, as described also for cows [24].

The end of lactation is a common time to administer antimicrobial therapy *to S. aureus* infected dairy cow because antibiotics can remain in the udder at higher concentrations for prolonged periods to effect a cure [25]. As described by Reeve-Johnson and Nickerson (2017), this microorganism is one of the most difficult to treat, especially during lactation, and there are no intramammary infusion products currently available that effectively eliminate *S. aureus* IMI from herds. During the present study, a reduction of SCM has been recorded in the second sampling compared to the first one in both the groups, although the difference was greater within the treated group than within the untreated one (*P* < 0.01 in the aDBT group vs. *P* < 0.05 in the no-aDBT group). Indeed, the survey showed encouraging results regarding the efficacy of the aDBT against SCM due to *S. aureus*. The results is confirmed by the very low RR value indicating a decreased risk of mastitis in MBs exposed to the antibiotic (RR = 0.06, *P <* 0.01), and by the clinical indexes showing higher *dry period cure rate* (aDBT group vs. no-aDBT group, *P <* 0.0001), *lower persistent mastitis rate* (aDBT group vs. no-aDBT group, *P <* 0.05) and *dry period new mastitis rate* (aDBT group vs. no-aDBT group, *P <* 0.01), in treated group than control one. Despite the potential beneficial effects observed, an assessment of the limitations may be represented by the chosen study design: indeed, the evaluation of the direct aDBT effects (without the attempt to estimate the overall population estimates), as well as the lack of information regarding the effect over-time may potentially produce an overestimation of the results observed. For these reasons, a further purpose is going to assess the effectiveness of aDBT on an entire MBs’ herd through a longitudinal study estimating the clinical outcomes entailed.

The non-lactating period can be considered a strategic moment for udder health because dry animals can recover from IMI present at drying-off [25]. Nevertheless, udder health can also be threatened as many cows acquire new IMI during the dry period [25]. This phenomenon may be considered the reason for the lack of efficacy against the appearance of new IMI due to *S. aureus* during dry-off. Regarding this point, the comparison with the results originating from cow medicine would be very difficult, since previous research on this topic aimed only to verified the effects of benzathine cloxacillin in animals with SCC > 200 × 103 cells/mL, and do not investigate the ability to prevent intramammary infection (i.e. infections do not producing a rise of SCC values above the threshold of normality) [5, 6]. In general, as reported for dairy species treated at dry-off [25–30], the effectiveness of a therapeutic protocol may be influenced several variables such as herd characteristics and management (e.g. prevalence of infected animals, lack of mastitis monitoring, etc.), length of dry-off (e.g. increased exposure to risk factors, etc.), biosecurity actions (e.g. farm’s hygiene protocols, etc.), type of treatment (e.g. administration route, posology, etc.) and lactation period (e.g. timing for sampling, etc.), that alone or interacting between them, can significantly influence the results observed.

According to the authors’ opinion, although aDBT showed an ability to mitigate the proportion of in-udder mono-infection due to *S. aureus*, two main factors may have adversely affected this parameter considered: strategies of herd management used during dry-off and length of this period. Indeed, the 16 weeks characterizing MBs’ dry-off period may increase the exposure time to risk factors of udder infections, regardless of whether they are treated or not [29]. Moreover, according to the label indications [21], this antibiotic should provide at least 7 weeks of protection against new infections after infusion, but its effectiveness during such a long dry-off period has been never studied, so far. Based on the previous considerations, the use of benzathine cloxacillin associated with excellent farm management practices may successfully improve the infection control within the herd at dry-off; therefore, further and more complete studies associated with suitable changes in management practices should be performed to verify the accuracy of the hypothesis.

As last, regarding the effects of aDBT on milk yield and quality, no significant inter-group difference was observed about daily mean production, while inter-and intra-group differences were detected for SCC. Regarding milk yield, it is widely described in literature that *S. aureus* can produce toxins destroying cell membranes and directly damaging milk-producing tissue in dairy cows [31, 32]. Indeed, during the infection, destruction of alveolar and ductal cells leads to the formation of scar tissue significantly limiting animals’ production potential [31, 32]. Likewise, MB affected by SCM at-dry-off may have developed structural damage of the secretive tissues negatively influencing the milk production at milk resuming and explaining the results observed. Instead, concerning the difference for SCC, the administration of aDBT may have reduced the severity pathological processes in progress generating the differences observed (Table 3). Moreover, as reported in Table 2, the significant reduction of SCM and a profitable *fresh calver mastitis rate* associated with a significant increase of negative quarters may also take part in the significant difference observed on overall SCC mean values. A further study purpose may be represented by the concomitant use of a teat sealant in pluriparous MB at drying-off to provide additional protection against the ingress of in-udder pathogens, contributing to prevent infections and mastitis during early lactation, as well as potentially reducing antibiotics use in these dairy ruminants.

## Conclusions

The clinical findings of the present investigation demonstrated for the first time the direct effects against *S. aureus* udder infection of antibiotic dry buffalo therapy based on benzathine cloxacillin. Even though a poor protective effect against new infection has been detected, likely due to a long dry-off period increasing the exposure time to risk factors for udder infections, the use of this antibiotic at drying-off seems to show encouraging results in reducing the proportion of positive MBs positive for *S. aureus*, the proportion of SCM due to the same pathogen, and the SCC values at the resumption of milking. Although this study was performed on a small number of animals, this investigation may be considered the starting point to build the essential basic knowledge promoting the increase of awareness necessary to understand the relevance of rational antibiotic uses in MB. The effects on the entire dairy herd under field conditions, as well as the in-depth analysis of the infectious dynamics, warrant further scientific exploration with the goal both to fully understand the effectiveness of benzathine cloxacillin in water buffalo and to create mastitis management programs ad-hoc developed for these dairy ruminants.

## Methods

The entire study was performed over two consecutive years. During the first one, the farm positive for *S. aureus* has been selected while during the second one the efficacy of the selected drug has been verified.

### Farm selection and management

The chosen farm was selected from a group of eight, located in Caserta district (Southern Italy), served by the local veterinary practitioner and monthly tested for bulk milk somatic cell count (BMSCC) and bacterial DNA by means of PCR-based assay as described in our previous study [5], between September 2016 and May 2017 (9-months). The eligibility criterion to select the farm of the study were represented by (i) presence of *S. aureus* within the milking herd, (ii) at least, the last 3, consecutive, bulk milk samplings positive for *S. aureus*. No strict criteria were instead applied for BMSCC or mastitis incidence. In the deseasonalized chosen farm of 400 dairy MBs, changes in herd management practices observed during the entire period of investigation (first and second year) were recorded to exclude possible influences on the following treatment protocol.

The selected farm was characterized by herringbone parlour and animals were milked twice a day. A 9-months average of 242 ± 27-day in milk (DIM), milk yield per head of 2420 kg and of BMSCC values of 256 × 10^3^ ± 37 × 10^3^ cell/mL has been recorded. Over the same period, the mean proportions of MBs infected (% herd currently infected, SCC > 200 × 10^3^ cell/mL) and chronically infected (% MB persistently infected, SCC > 200 × 10^3^ cell/mL for two of the previous three consecutive tests) were 39.8% ± 6.8 (±SD) and 22.2% ± 4.6, respectively. Regarding dried-off MBs, all of them were kept in a common paddock of ~ 600 m^2^ (~ 15 m × 40 m). The barn was characterized by a roofed common bedded, exercise areas and feeding alley, all of them characterized by solid non-grooved concrete floor (cleaned once a day). As a common practice in MB herd management, animals were abruptly dried off when they were producing < 0.5 L/day [5]. Dry animals were fed with a total mixed ration including hay, silage, and a multi-vitamin integrator two times a day; free access to the protected water trough was always guaranteed.

### Animal enrolment and therapy

During the second year of study, 40 of 108 (37.0%) pluriparous MBs (≥2 lactations), has been selected by means convenient sampling. These represent all pluriparous having the following characteristics: dried-off between June and September 2018, in good health, having 4 functional quarters, teats free of significant lesions and without obvious clinical signs of mastitis recorded during at least the last 3 months before dry-off.

The sample population selected has been randomly divided into aDBT group (made of 20 MBs receiving therapy at drying-off) and no-aDBT group (made of 20 MBs left untreated) consisting of 80 quarters each one (overall 160 quarters). At drying-off, all the animals enrolled were individually submitted to a complete clinical examination with particular focus on the teat and udder health status [10] and received the first quarter milk sampling. At this time, the aDBT group received an intramammary administration for each quarter of 600 mg of benzathine cloxacillin in 3.6 g of suspension (Orbenin Extra®, Zoetis, Inc.), while the no-aDBT group was left untreated. Antibiotic dry therapy was performed according to Skidmore et al. [33]. Briefly: (i) milking the buffalo completely, utilizing proper milking technique; (ii) cleaning teats with a clean and dry towel; (iii) pre-dipping all 4 teats with effective teat disinfectant; (iv) leaving the animal alone 5–10 min; (v) pre-dipping again; (vi) infuse antibiotics by introducing the tip only 5 mm into the teat canal; (vii) post-dipping all teats and keep the buffalo restrained for another hour to facilitate the closing of the teat opening and prevent the introduction of bacteria into the exposed teat opening. Moreover, all MB were daily monitored for milk leakage, during the first week after drying-off and in the last week before the estimated day of calving. One every second week, animals were also monitored for swollen quarters and teat damage during the entire dry period.

As suggested by Bradley et al. [26], to gain an insight into the likely origin of mastitis and the importance of treatment in mastitis epidemiology the second quarter milk sampling has been performed on all MBs (aDBT and no-aDBT groups) within 10 DIM of new lactation (range: 7–10 DIM, median: 9.0 DIM).

All quarters milk samples were aseptically collected as described by the National Mastitis Council [34] for dairy cows. Milk collection has been performed immediately before regular evening milking, in double aliquots and using sterile test tubes (BD Vacutainer, Oxford, UK). For all of them, bacteriological milk culture (BMC) and SCC investigations were performed to provide information about *S. aureus* presence and severity of the disorder; finally, daily milk yields were also recorded during the same samplings time using automatic dedicated software (Afifarm, Afimilk, Kibbutz Afikim, Israel).

All the milk samplings were performed during the mastitis monitoring program regularly practiced by the local veterinary practitioner (e.g. including regular SCC monitoring, data analysis, antibiotic susceptibility testing before treatments, etc.). All MB belonging to aDBT groups were infected by *S. aureus* susceptible to benzathine cloxacillin and other antibiotics belonging to penicillin group (i.e. ampicillin, oxacillin, penicillin G). All actions performed by the investigators abide by the *common good clinical practices* [35]. Moreover, the farmer was previously informed and in agreement with purposes as well as methods used.

### Cytological and bacteriological examination

The examinations were performed according to the diagnostic procedures used in our previous studies [1, 4, 6]. Briefly, milk samples were placed in a cool box (4 °C) and brought to the reference laboratory within 1 h (h) of collection. Samples were submitted to SCC analysis (Fossomatic 5000, Foss Electric, Hillerod, Denmark, approved for buffalo) and BMC within 2 h from collection. The BMC and bacteriological identification were performed according to the National Mastitis Council guidelines [36]. All of them were performed without previous information about the udder’s clinical status and in double (to confirm BMC results and to assign the correct bacteriological positivity). Briefly, 10 μL of each milk sample was streaked on one-quarter of a blood-agar plate (Merck KGaA, Darmstadt, Germany), incubated at 37 °C for up to 48 h and examined two times (at 24 and 48 h of incubation). Bacterial colonies were tentatively identified on gross morphology. The number and types of colonies were also recorded. As described in previous studies [4, 6, 10], when 3 or more dissimilar colony types were isolated on the plate, the sample was considered contaminated. Gram staining and catalase testing were performed to differentiate between streptococci and staphylococci, and tube coagulase testing using rabbit plasma to differentiate between coagulase-positive and coagulase-negative staphylococci. A final identification of microorganisms was performed using the colorimetric and automatic identification system (Vitek 2 XL 120; bioMerieux Inc., Hazelwood, MO). Enterobacteriaceae have been grown on MacConkey agar (Oxoid, Basingstoke, UK) and identified using the same automatic system. Mastitogens bacteria with confidence levels greater than 0.90 were considered identified at the species level; otherwise, they were identified at the genus level. One CFU/mL was the cut-off to assign the positivity for *S. aureus*.

### Clinical definition of udder health status

As reported in our previous studies [5, 6], different udder health status has been classified according to SCC values, microbiological status and clinical signs of disease. Briefly, quarters producing milk with SCC > 200 × 10^3^ cells/mL and positive BMC have been classified as affected by mastitis, with (clinical mastitis, CM) or without clinical signs (subclinical mastitis, SCM). Quarters producing milk with SCC < 200 × 10^3^ cells/mL but positive BMC caused by udder specific-pathogens (i.e. belonging to the contagious, environmental or opportunistic categories) have been instead classified as affected by IMI. Finally, quarters with SCC < 200 × 10^3^ cells/mL and negative BMC were defined as negative.

### Statistical analysis

For the present investigation, only data originating from quarters considered as affected by CM, SCM and IMI due to *S. aureus* were analyzed and discussed.

Different quarter’s health status, SCC, and milk yields were analyzed by standard descriptive statistics, while normality has been assessed by means of Shapiro Wilk tests, normal probability plots, and histograms was assessed. Data were expressed as absolute numbers, percentages, or mean ± SD. Log-transformation was applied to variable not normally distributed.

As reported by Bradley et al. [26], several clinical indexes were calculated to verify antibiotic’s clinical efficacy against *S. aureus* mastitis, comparing intra-group (drying-off vs. < 10 DIM) and inter-group differences (aDBT vs. no-aDBT, at both the samplings). They included: (i) *fresh calver mastitis rate* (as the overall proportion of quarters affected by CM or SCM due to *S. aureus* within 10 DIM); (ii) *dry period new mastitis rate* (proportion of quarters affected by IMI due to *S. aureus* at dry-off, but developing CM or SCM within 10 DIM); (iii) *dry period cure rate* (proportion of quarters affected by CM or SCM due to *S. aureus* at dry–off that become affected by IMI or negative within 10 DIM); (iv) *persistent mastitis rate* (proportion of quarters affected by CM or SCM due to *S. aureus* at dry-off and still sick within 10 DIM). Proportion of quarters with CM, SCM, and IMI due to *S. aureus* at drying-off and milking resumption were also calculated. Instead, the proportion of animal infected was assessed considering all MBs with at least one positive quarter for *S. aureus* (i.e. affected by CM, SCM or IMI). For each MB belonging to the aDBT group, the number of quarters affected by mastitis has been also calculated pre- and post-treatment to assess the therapy effectiveness depending on the number of quarters affected per animal. Log-transformed (SCC) and untransformed (milk yields) continuous variables were compared using simple Student’s *t*-test. Significant differences between expected and observed frequencies of categorical data (e.g. clinical indexes for aDBT monitoring) were assessed using χ2 –test’s contingency tables. Fisher’s exact test was performed in case of low expected frequencies (< 5). Moreover, the ratio of the probability that mastitis (CM and SCM) might occur in aDBT group (exposed group) at the resumption of milking, versus the probability that it might appear in no-aDBT (non-exposed group) has been quantified by means of Relative Risk (RR) and 95% confidence intervals calculated according to Petrie and Watson [37].

*P*robabilities < 0.05 were considered statistically significant. All the statistical data were analyzed using dedicated software (SPSS, Version 17.0, Chicago, IL, USA).

## Data Availability

The datasets used and/or analysed during the current study are available from the corresponding author on reasonable request.

## Abbreviations

aDBT: antibiotic dry buffalo therapy
BMC: Bacteriological milk culture
BMSCC: Milk somatic cell count
CI: Confidence interval
CM: Clinical mastitis
CNS: Coagulase negative staphylococci
DIM: Days in milk
IMI: Intramammary infections
MBs: Mediterranean buffaloes
no-aDBT: No antibiotic dry buffalo therapy
SCC: Somatic cell count
*S. aureus*: *Staphylococcus aureus*
SD: Standard deviation
SCM: Subclinical mastitis
RR: Relative risk
